# Oxidative stress and DNA damage profile in cord blood of vitamin B12-deficient newborns

**DOI:** 10.5937/jomb0-60757

**Published:** 2026-02-27

**Authors:** Dogan Kose, Mahmut Demir, Adnan Kirmit

**Affiliations:** 1 Harran University, Faculty of Medicine, Department of Paediatric Haematology and Oncology, Sanliurfa, Turkey; 2 Harran University, Faculty of Medicine, Department of Paediatrics, Sanliurfa, Turkey; 3 Harran University, Faculty of Medicine, Department of Biochemistry, Sanliurfa, Turkey

**Keywords:** 8-hydroxy-2'-deoxyguanosine, DNA damage, newborn, oxidative stress, vitamin B12, 8-hidroksi-2'-deoksiguanozin, oštećenje DNK, novorođenče, oksidativni stres, vitamin B12

## Abstract

**Background:**

Vitamin B12 is an essential biomolecule involved in DNA synthesis, epigenetic regulation, and energy metabolism. Maternal vitamin B12 deficiency during pregnancy has been linked to oxidative stress and impaired DNA integrity. This study aimed to investigate oxidative stress and DNA damage biomarkers in term newborns with vitamin B12 deficiency and to assess their diagnostic value.

**Methods:**

A single-centre, prospective case-control study was conducted in 48 term newborns with vitamin B12 deficiency and 40 matched healthy controls. Cord venous blood samples were analysed for vitamin B12, oxidative stress parameters, and a DNA damage marker. Diagnostic performance was evaluated by receiver operating characteristic analysis.

**Results:**

Vitamin B12 levels were markedly lower in the patient group than in controls. Newborns with vitamin B12 deficiency had significantly higher levels of 8-hydroxy-2'-deoxyguanosine, total oxidant status, and oxidative stress index, whereas total antioxidant status did not differ. Receiver operating characteristic analysis demonstrated the highest diagnostic accuracy for total oxidant status, followed by 8-hydroxy-2'-deoxyguanosine and oxidative stress index.

**Conclusions:**

Oxidative stress and DNA damage were clearly increased in vitamin B12-deficient newborns. These findings suggest that maternal vitamin B12 deficiency leaves detectable biochemical imprints at birth. This prospective analysis highlights that total oxidant status, oxidative stress index, and 8-hydroxy-2'-deoxyguanosine may serve as valuable diagnostic biomarkers and, to our knowledge, represents one of the few studies linking vitamin B12 deficiency to oxidative damage in newborns.

## Abstract (sr)

**Uvod:**

Vitamin B12 je esencijalni biomolekul koja učestvuje u sintezi DNK, epigenetskoj regulaciji i metaboličkim procesima energije. Deficit vitamina B12 kod majke tokom trudnoće povezan je sa oksidativnim stresom i narušenim integritetom DNK. Cilj ove studije bio je da se ispita oksidativni stres i biomarkeri oštećenja DNK kod novorođenčadi sa deficitom vitamina B12 i da se proceni njihova dijagnostička vrednost.

**Metode:**

Sprovedena je prospektivna studija slučaj-kontrola u jednoj ustanovi koja je obuhvatila 48 novorođenčadi u terminu sa deficitom vitamina B12 i 40 odgovarajućih zdravih kontrola. Uzorci venske krvi iz pupčane vrpce analizirani su na nivo vitamina B12, parametre oksidativnog stresa i markere oštećenja DNK. Dijagnostička efikasnost procenjena je analizom ROC krive.

**Rezultati:**

Nivoi vitamina B12 su bili značajno niži u grupi pacijenata u odnosu na kontrolnu grupu. Novorođenčad sa deficitom vitamina B12 je imala značajno više nivoe 8-hidroksi-2'-deoksiguanozina, ukupnog oksidativnog statusa i indeksa oksidativnog stresa, dok ukupni antioksidativni status nije pokazao razlike. Analiza ROC krive je pokazala najveću dijagnostičku tačnost za ukupni oksidativni status, zatim za 8-hidroksi-2'-deoksiguanozin i indeks oksidativnog stresa.

**Zaključak:**

Oksidativni stres i oštećenje DNK su bili jasno povećani kod novorođenčadi sa deficitom vitamina B12. Ovi nalazi sugerišu da deficit vitamina B12 kod majke ostavlja detektabilne biohemijske tragove već pri rođenju. Ova prospektivna analiza ističe da ukupni oksidativni status, indeks oksidativnog stresa i 8-hidroksi-2'-deoksiguanozin mogu da posluže kao značajni dijagnostički biomarkeri, a prema našem saznanju, predstavlja jednu od retkih studija koja povezuje deficit vitamina B12 sa oksidativnim oštećenjem kod novorođenčadi.

## Introduction

The neonatal period not only marks the beginning of postnatal life but also constitutes a critical stage that shapes neurodevelopmental processes and metabolic programming in the years that follow [1]. Micronutrient deficiencies that occur during this vulnerable period may affect epigenetic regulatory mechanisms, mitochondrial energy production, and cellular redox balance, thereby predisposing to long-term, irreversible biological alterations [2].

Vitamin B_12_ (B_12_) is an essential vitamin that the human body cannot synthesise, is water-soluble, and must be obtained from external animal-based dietary sources [3]. It is a vital cofactor involved in DNA synthesis, the methylation cycle, methionine production, homocysteine metabolism, hematopoiesis, and protein synthesis [3][4].

B_12_ is also essential for myelin synthesis and nervous system functions [3]. During the first six months of life, the central nervous system undergoes rapid structural and functional maturation, including myelination. B_12_ deficiency during this period may lead to irreversible neurological damage, developmental delays in cognition and motor function, neuromotor disorders, and permanent neurological sequelae [2][4]. Animal studies have shown that maternal B_12_ deficiency adversely affects brain development by reducing neuronal branching density and synaptic connectivity [5].

B_12_ deficiency also affects mitochondrial functions and epigenetic control mechanisms [2]. In newborns, this condition is often assessed through homocysteine and methylmalonic acid levels, and it has been reported to be closely associated with fetal growth, birth weight, and early neurodevelopment [3]. The increase in these biomarkers may lead to reduced ATP production [6] and to cellular damage triggered by increased mitochondrial-derived reactive oxygen species [7][8].

One of the specific indicators of oxidative DNA damage is 8-hydroxy-2'-deoxyguanosine (8-OHdG), which is formed as a result of oxidative modification of the guanine base. This molecule is considered a reliable biomarker for assessing the effects of oxidative stress at the cellular and genetic levels [9].

Studies investigating the effects of B_12_ deficiency on oxidative stress profile and DNA damage in newborns are quite limited [10]. Clinical studies evaluating this relationship through 8-OHdG levels in childhood are also rare, and the available data are mostly confined to adult populations or in vitro studies [7].

In this study, oxidative stress markers (total oxidant status; TOS, total antioxidant status; TAS, oxidative stress index; OSI) and the DNA damage marker (8-OHdG) were evaluated together in term newborns with low B_12_ levels at birth; in addition, the diagnostic performance of these parameters in predicting B_12_ deficiency was analysed using receiver operating characteristic (ROC) analysis. The research aimed to demonstrate the biochemical stress experienced during the intrauterine period through cord blood and to identify the most reliable biomarkers for clinical diagnosis.

## Materials and methods

### Ethics

This study was conducted in accordance with the principles of the Declaration of Helsinki. It was approved by the Ethics Committee of Scientific Research of the Faculty of Medicine, Harran University (approval date: July 13, 2017; approval number: 07). Written informed consent was obtained from the parents of all participating newborns.

### Study groups

The research was designed as a single-centre, prospective case-control study. The study group consisted of 48 term newborns born at 38-42 weeks of gestation, in good general health but diagnosed with B_12_ deficiency. The control group included 40 healthy newborns born within the same gestational age range. All deliveries were performed at the Obstetrics and Gynaecology Clinic of Harran University.

### Exclusion criteria

The following conditions were excluded from the study: acute infection, multiple pregnancy, birth weight < 2500 g, congenital anomaly, need for intervention during delivery, requirement for oxygen support, Apgar score < 8, need for neonatal intensive care, jaundice, phototherapy, asphyxia, oligohydramnios, polyhydramnios, preterm labor, placenta previa, placental abruption, preeclampsia, maternal chronic disease, infection during pregnancy, history of smoking or alcohol consumption, and anemia. The criteria were determined based on maternal medical records, delivery notes, and neonatal examination findings.

### Collection and storage of blood samples

Umbilical cord blood is a reliable biological sample that reflects the fetus's nutritional and metabolic status during the intrauterine period and is widely used in neonatal research [3]. Biomarkers obtained from these samples provide an opportunity to evaluate maternal-fetal transfer and the redox status at birth. During delivery, venous blood samples were obtained from the umbilical vein of the placental segment immediately after cord clamping in cases meeting the criteria. All cord blood samples were collected following early cord clamping (within 30 seconds after delivery) to ensure consistency among participants. The samples were centrifuged at 3500 rpm for 10 minutes to separate the sera. Serum B_12_ levels were measured first; those with deficiency were included in the study group, while those with normal values were included in the control group. The remaining serum samples were stored at -80°C for analysis of 8-OHdG, TOS, and TAS. All samples were non-hemolysed, and a single freeze-thaw cycle was applied before biochemical analyses. The time between sample collection and initial processing did not exceed 30 minutes.

### B_12_ measurement

Serum B_12_ levels were determined using the chemiluminescent microparticle immunoassay method on the Architect i2000SR analyser (Abbott Diagnostics, Abbott Park, IL, USA). The measurement range was 150-2000 pg/mL, with an analytical sensitivity of <5%. Results were reported as picograms per millilitre (pg/mL). In accordance with neonatal reference ranges, vitamin B_12_ deficiency was defined as a serum concentration of <200 pg/mL, as reported in previous pediatric and perinatal studies.

### TOS measurement

Oxidative stress occurs when the oxidant load exceeds the antioxidant defence capacity. TOS is a comprehensive indicator of systemic oxidant capacity [11]. Serum TOS levels were measured using commercial kits (Rel Assay Diagnostics, Gaziantep, Turkey) by the colourimetric method. The method is based on the principle that oxidant compounds oxidise ferrous ions to ferric ions. Results were expressed as micromoles of hydrogen peroxide (H_2_O_2_) equivalent per litre (μmol H_2_O_2_ eq/L) [11].

### TAS measurement

Total antioxidant capacity was assessed using the Trolox equivalent antioxidant capacity (TEAC) method, expressed in this study as total antioxidant status (TAS). TAS reflects the total antioxidant capacity of plasma [12]. Serum TAS levels were determined using commercial kits. The measurement is based on the colour change that occurs when antioxidants reduce the 2,2'-azino-bis(3-ethylbenzothiazoline-6-sulfonic acid) radical cation. Trolox was used for calibration. Results were reported as millimoles of Trolox equivalent per litre (mmol Trolox eq/L) [12]. For each assay, internal quality control was performed using the calibrators and control sera supplied with the commercial kits to ensure inter-assay consistency.

### OSI calculation

OSI is a parameter reflecting the degree of oxidative stress and is calculated as the TOS/TAS ratio [12]. To equalise measurement units, the TAS value was multiplied by 10. Results were expressed in arbitrary units (AU) [11]. OSI (AU) = [TOS (μmol H_2_O_2_ eq/L) / TAS (mmol Trolox eq/L)] X 10.

### Measurement of 8-OHdG levels

Serum 8-OHdG levels were measured by enzyme-linked immunosorbent assay (ELISA) using a commercial kit (Cayman Chemical, catalogue no. 589320; Ann Arbour, MI, USA). Samples were diluted 1:50 according to the manufacturer's instructions. Assay sensitivity was 33 pg/mL (lowest detectable concentration). The intra-assay coefficient of variation was 4.7-11.6% and the inter-assay coefficient of variation was 4.5-10.7%. Results were reported in ng/mL [13].

### Statistics

Data were analysed using NCSS 2007 software (Number Cruncher Statistical System; Kaysville, Utah, USA). Descriptive statistics were presented as mean, standard deviation (SD), median, minimum, maximum, frequency, and percentage. Normality was assessed by Kolmogorov-Smirnov and Shapiro-Wilk tests along with graphical inspections. For comparisons between two groups, the independent-samples Student's t-test was used when data were normally distributed, and the Mann-Whitney U test was used when data were not normally distributed. Categorical variables were analysed using Pearson's chi-square test. The predictive power of biomarkers for B_12_ deficiency was evaluated using ROC curve analysis. Cut-off values were determined by calculating sensitivity (Sens), specificity (Spec), positive predictive value (PPV), negative predictive value (NPV), and area under the curve (AUC). AUC values were used to assess the test's discriminative performance, and cut-off points were identified using the Youden index. All analyses were two-tailed, and P<0.05 was considered statistically significant. No formal power analysis was performed; the sample size was determined based on the availability of eligible participants during the study period.

## Results

Of the 48 newborns diagnosed with B_12_ deficiency, 62.5% (n = 30) were female and 37.5% (n = 18) were male. In the control group of 40 newborns, 67.5% (n = 27) were female and 32.5% (n = 13) were male. The mean gestational age was 39.31 ±0.95 weeks in the patient group and 39.25±1.01 weeks in the control group. The mean birth weight was 3234.06±293.71 g in the patient group and 3262.25±254.94 g in the control group. Birth length was 50.00±1.27 cm and 49.93±1.38 cm, and head circumference was 36.46±1.32 cm and 36.53±1.15 cm, respectively. In the patient group, 27.1% (n = 13) of the deliveries were cesarean section and 72.9% (n = 35) were normal spontaneous vaginal delivery, whereas in the control group these rates were 22.5% (n = 9) and 77.5% (n = 31), respectively. No statistically significant differences were found between the groups for sex, gestational age, birth weight, birth length, head circumference, or mode of delivery (Table 1).

**Table 1:** Comparison of gestational age, anthropometric measurements, gender, and mode of delivery between the patient and control groups. C/S: Cesarean section; Max: maximum; Min: minimum; NSVD: normal spontaneous vaginal delivery; SD: standard deviation.

		Patient group<br>(n=48)	Control group<br>(n=40)	P
Birth (weeks)	Min-Max (Median)	37-40 (40)	37-40 (40)	0.765
	Mean ± SD	39.31±0.95	39.25±1.01	
Weight (g)	Min-Max (Median)	2780-3900 (3230)	2750-3700 (3270)	0.636
	Mean ± SD	3234 .06 ± 293 .71	32 62 .25 ± 254 .94	
Length (cm)	Min-Max (Median)	48-52 (50)	47-52 (50)	0.792
	Mean ± SD	50.00±1.27	49.93±1.38	
Head circumference (cm)	Min-Max (Median)	34-38 (37)	34-38 (37)	0.804
	Mean ± SD	36.46±1.32	36.53±1.15	
Gender; n (%)	Female	30 (62.5)	27 (67.5)	0.625
	Male	18 (37.5)	13 (32.5)	
Mode of delivery; n (%)	C/S	13 (27.1)	9 (22.5)	0.621
	NSVD	35 (72.9)	31 (77.5)	

**Figure 1:**
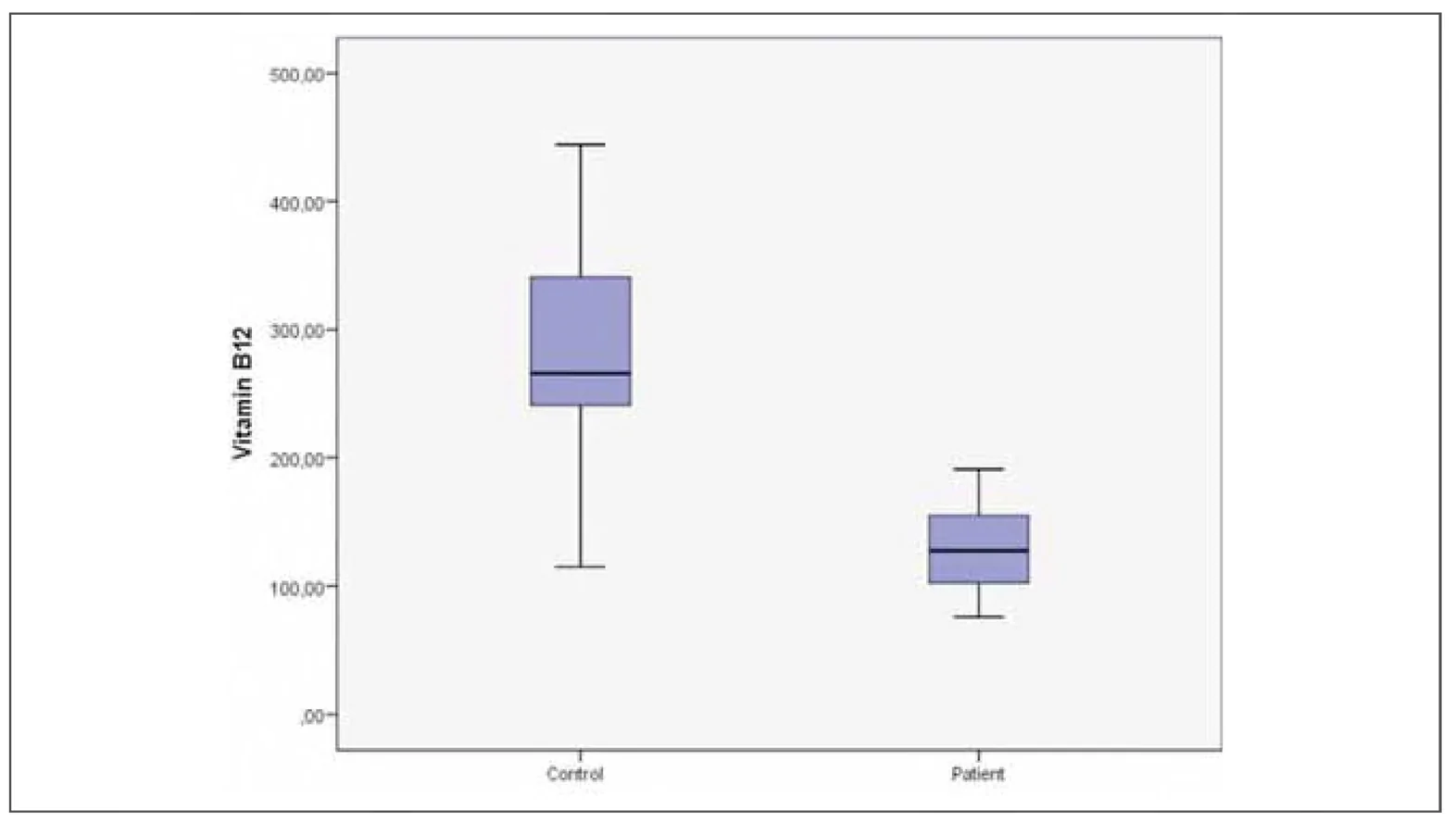
Comparison of patient and control groups regarding serum vitamin B_12_ levels.

Serum B_12_ level was 128.06±31.89 pg/mL in the patient group and 280.90±64.45 pg/mL in the control group, being significantly lower in the patient group (P=0.001) (Table 2) (Figure 1).

**Table 2:** Comparison of vitamin B_12_, 8-OHdG, total antioxidant status, total oxidant status, and oxidative stress index values between the study groups. Vitamin B_12_ values are expressed in pg/mL; 8-OHdG in ng/mL; TAS in mmol Trolox eq/L; TOS in mmol H_2_O_2_ eq/L; and OSI in arbitrary units (AU).<br>8-OHdG: 8-hydroxy-2'-deoxyguanosine; AU: Arbitrary units; Max: Maximum; Min: Minimum; OSI: Oxidative stress index; SD: Standard deviation; TAS: Total antioxidant status; TOS: Total oxidant status; Trolox eq: Trolox equivalents; H_2_O_2_ eq: Hydrogen peroxide equivalents; B_12_: Vitamin B_12_.

		Patient group(n=48)	Control group (n=40)	P
B_12_ (pg/mL)	Min-Max (Median)	76-191 (127.5)	115-444 (266)	0.001
	Mean ± SD	128.06±31.89	280.90±64.45	
8-OHdG (ng/mL)	Min-Max (Median)	9.1-28.3 (12)	0.7-15.3 (9.7)	0.001
	Mean ± SD	12.94±3.29	9.04±3.92	
TAS (mmol Trolox eq/L)	Min-Max (Median)	0.1-1.5 (1.1)	0.3-2.5 (1)	0 .904
	Mean ± SD	0.98±0.36	0.99±0.42	
TOS (μmol H_2_O_2_ eq/L)	Min-Max (Median)	3.8-25.5 (23.4)	0.9-24.9 (13)	0.001
	Mean ± SD	21.07±5.31	12.59±8.86	
OSI (AU)	Min-Max (Median)	12.7-134.8 (21.7)	1-57.8 (13.8)	0.001
	Mean ± SD	26.50±18.81	15.49±13.15	

**Figure 2:**
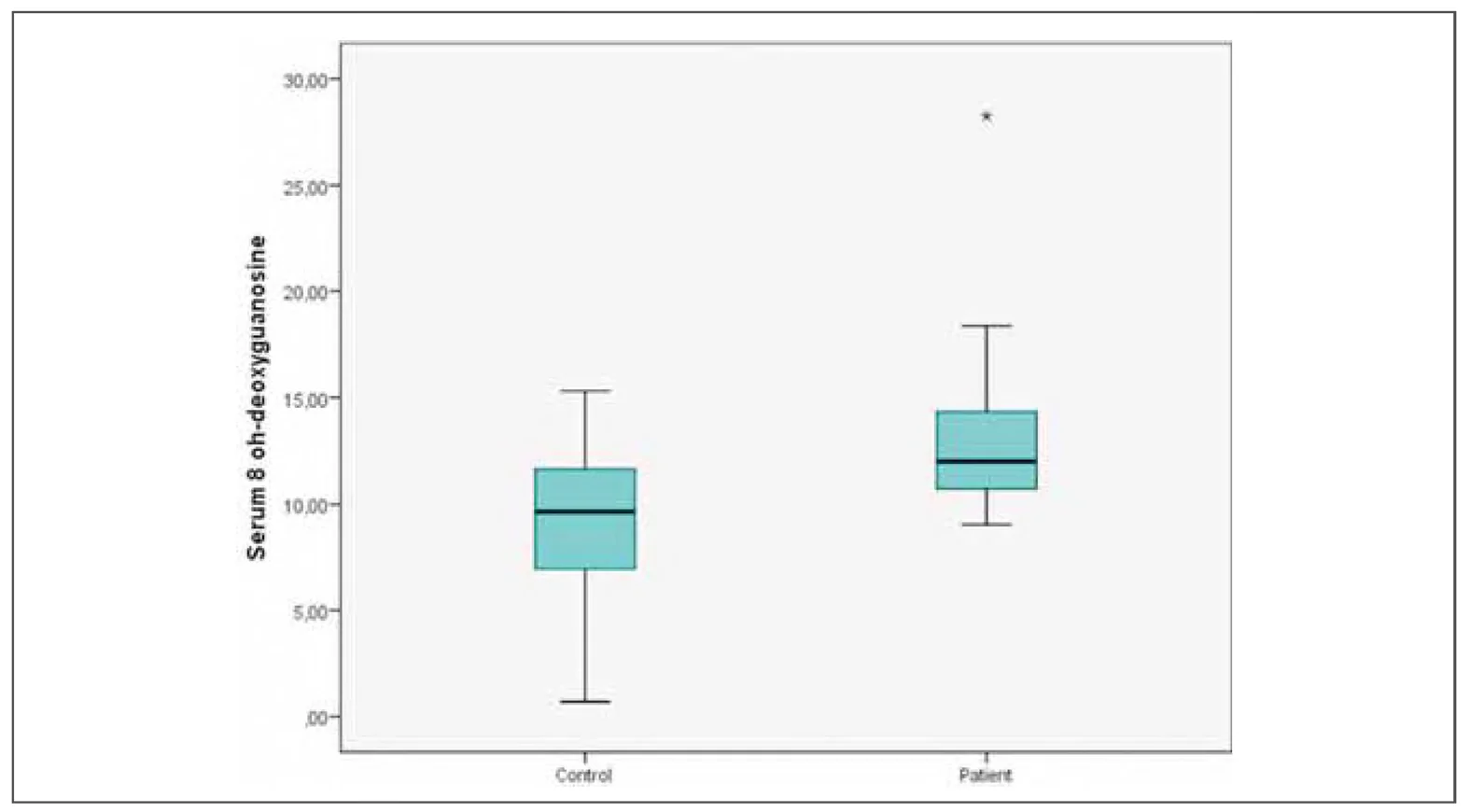
Comparison of patient and control groups regarding serum 8-OHdG levels (8-OHdG, 8-hydroxy-2'-deoxyguanosine).

Serum 8-OHdG level was 12.94±3.29 ng/mL in the patient group and 9.04 ± 3.92 ng/mL in the control group, and it was significantly higher in the patient group (P=0.001) (Table 2) (Figure 2).

**Figure 3:**
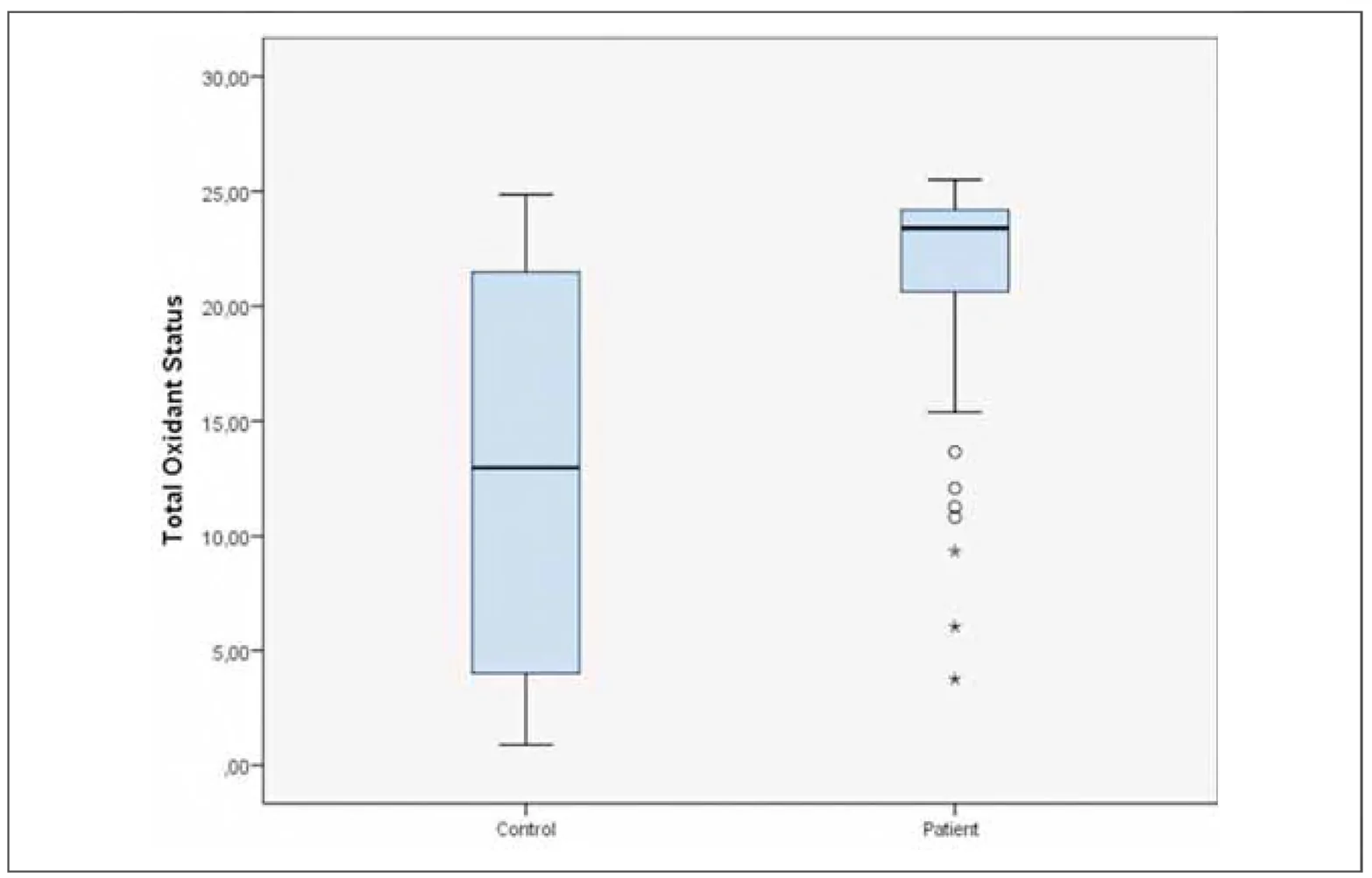
Comparison of patient and control groups regarding total oxidant status (TOS, total oxidant status).

The mean TOS was 21.07±5.31 μmol H_2_O_2_ eq/L in the patient group and 12.59 ± 8.86 μmol H_2_O_2_ eq/L in the control group, being significantly higher in the patient group (P=0.001) (Table 2) (Figure 3).

The mean TAS was 0.98±0.36 mmol Trolox eq/L in the patient group and 0.99±0.42 mmol Trolox eq/L in the control group, with no significant difference between the two groups (P=0.904) (Table 2).

**Figure 4:**
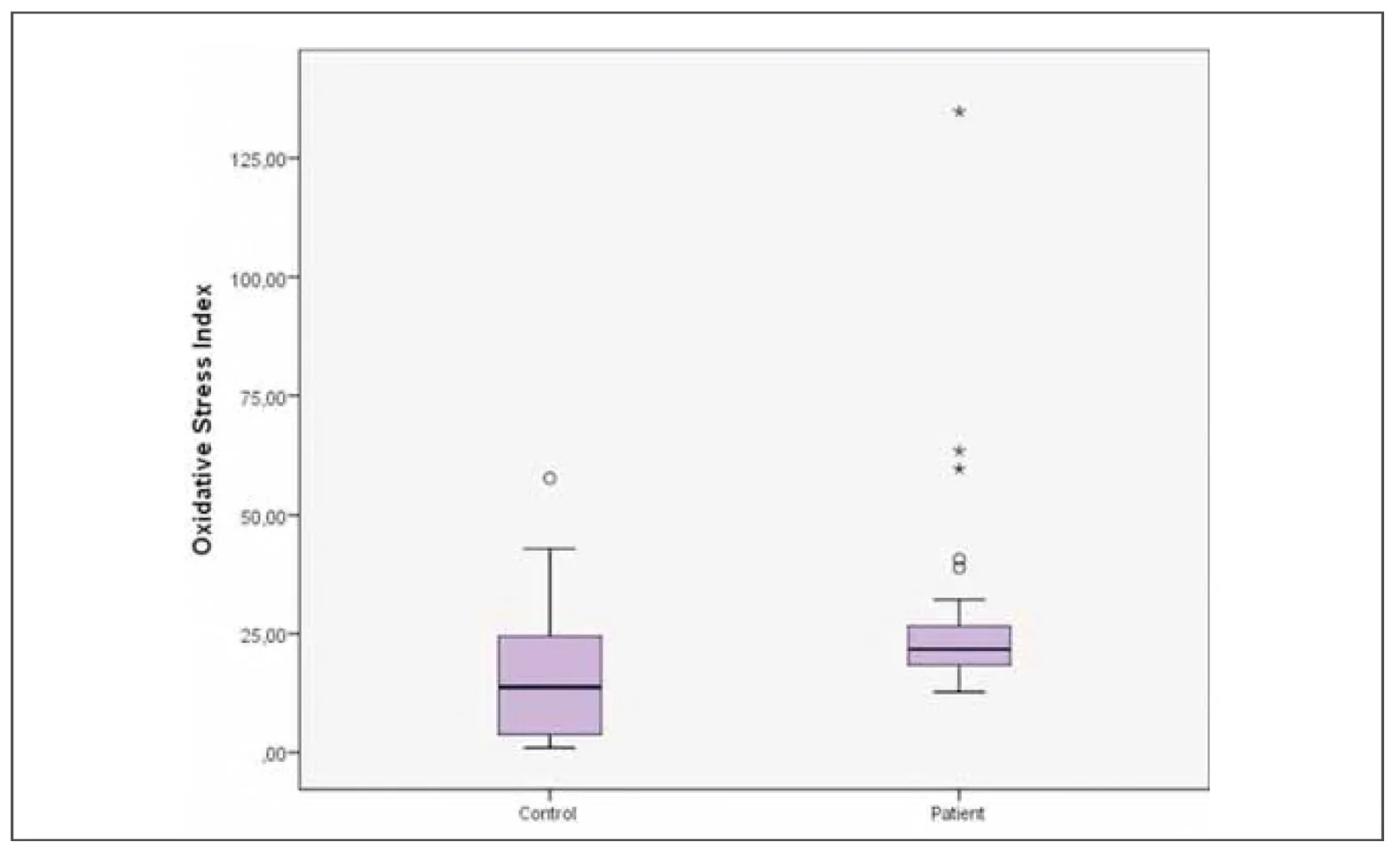
Comparison of patient and control groups regarding oxidative stress index (OSI, oxidative stress index).

The OSI was calculated as 26.50±18.81 AU in the patient group and 15.49±13.15 AU in the control group, being significantly higher in the patient group (P=0.001) (Table 2) (Figure 4).

**Figure 5:**
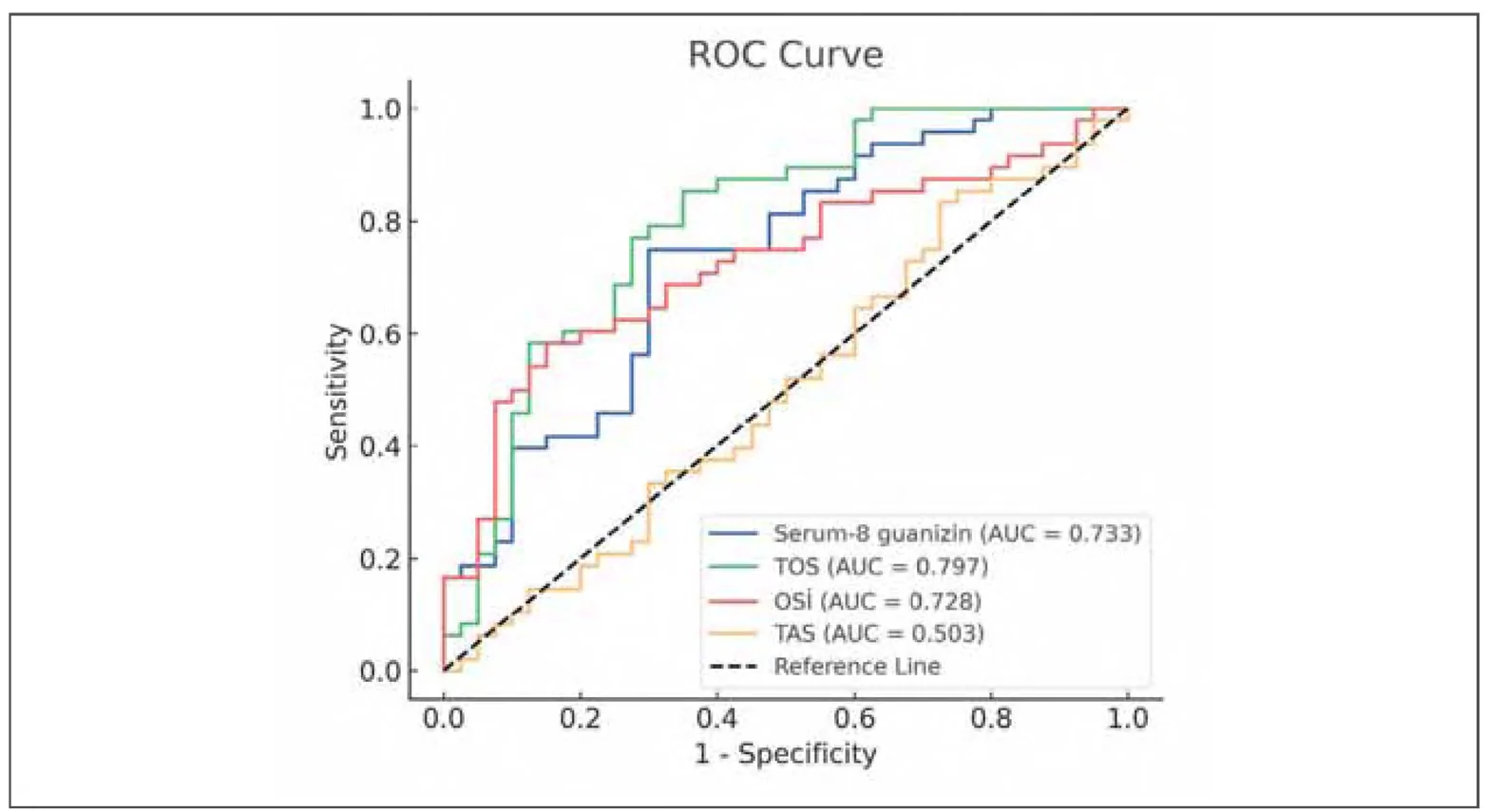
ROC curves for serum 8-OHdG, TOS, OSI, and TAS in predicting vitamin B_12_ deficiency in newborns (8-OHdG, 8-hydroxy-2';-deoxyguanosine; TOS, total oxidant status; OSI, oxidative stress index; TAS, total antioxidant status).

In the ROC analysis, the diagnostic values of serum 8-OHdG, TAS, TOS, and OSI levels in predicting B_12_ deficiency were evaluated. When the cut-off value for serum 8-OHdG was set at ≥9.72 ng/mL, sensitivity was 95.83%, specificity was 52.50%, PPV was 70.77%, and NPV was 91.30% (AUC=0.758; 95% CI: 0.656-0.860; P=0.001). For TAS, at a cut-off value of ≥0.27 mmol Trolox eq/L, sensitivity was 100.00%, specificity was 7.50%, PpV was 55.17%, and NPV was 100.00% (AUC = 0.409; P>0.05). For TOS, at a cut-off value of ≥20.06 μmol H_2_O_2_ eq/L, sensitivity was 79.17%, specificity was 67.50%, PPV was 74.51%, and NPV was 72.97% (AUC=0.802; 95% CI: 0.710-0.894; P=0.001). For OSI, at a cut-off value of ≥17.7, sensitivity was 85.42%, specificity was 60.00%, PPV was 71.93%, and NPV was 77.42% (AUC = 0.730; 95% CI: 0.616-0.844; P=0.001) (Table 3) (Figure 5).

**Table 3:** Diagnostic performance of serum 8-OHdG, total antioxidant status, total oxidant status, and oxidative stress index in predicting vitamin B12 deficiency. 8-OHdG values are expressed in ng/mL; TAS in mmol Trolox eq/L; TOS in mmol H_2_O_2_ eq/L; and OSI in arbitrary units (AU).<br>8-OHdG: 8-hydroxy-2'-deoxyguanosine; AUC: area under the curve; AU: arbitrary units; CI: Cconfidence interval; μmol: micromole; mmol: millimole; NPV: negative predictive value; OSI: oxidative stress index; PPV: positive predictive value; Sens: sensitivity; Spec: specificity; TAS: total antioxidant status; TOS: total oxidant status; Trolox eq: trolox equivalents; H_2_O_2_ eq: hydrogen peroxide equivalents.

	Cut-off	Sens (%)	Spec (%)	PPV (%)	NPV (%)	AUC	95% CI	P
8-OHdG<br>(ng/mL)	≥9.72	95.83	52.50	70.77	91.30	0.758	0.656-0.860	0.001
TAS (mmol<br>Trolox eq/L)	≥0.27	100.00	7.50	55.17	100.00	0.409	0.286-0.532	0.136
TOS (μmol<br>H_2_O_2_ eq/L)	≥20.06	79.17	67.50	74.51	72.97	0.802	0.710-0.894	0.001
OSI (AU)	≥17.7	85.42	60.00	71.93	77.42	0.730	0.616-0.844	0.001

## Discussion

B_12_ plays a critical role in the methionine synthase reaction, which is involved in DNA methylation and synthesis. This process is a fundamental step in regulating cellular proliferation and gene expression [6]. The reaction product, tetrahydrofolate, is used in the synthesis of thymidine. In cases of deficiency, these mechanisms are disrupted; uracil is incorporated into DNA in place of thymidine, leading to genetic damage such as point mutations and strand breaks [14].

One of the key biomarkers of DNA damage, 8-OHdG, is formed as a result of oxidative modification of the guanine base. It reflects both base-level changes in DNA and the organism's overall oxidative stress burden [15]. During DNA repair, 8-OHdG is excised and released into the bloodstream and urine, serving as an indirect indicator of systemic oxidative DNA damage [16]. It has also been reported that 8-OHdG levels can be measured in newborns shortly after birth [17].

In our study, the finding that serum 8-OHdG levels were significantly higher in newborns with B_12_ deficiency than in the control group suggests that DNA damage may occur even when B_12_ deficiency begins in utero. It has been reported that 8-OHdG levels measured in the first postnatal months of very low birth weight infants are associated with mental development at 18 months [18]. Therefore, 8-OHdG levels measured at birth may have prognostic value.

Previous studies have reported that TOS and OSI levels are elevated, whereas TAS levels are reduced in cases of B_12_ deficiency [19]. In our findings, TOS and OSI were significantly higher, whereas TAS was similar to that of the control group. This highlights the importance of vitamin B_12_ in maintaining redox balance during the neonatal period. In deficiency states, the increased oxidant burden overwhelms the defence mechanisms, leading to stress. Furthermore, the fact that antioxidant systems are not yet fully mature in newborns [7][20] and that enzymatic antioxidant levels have not reached adult levels may explain why no significant changes were observed in TAS levels [7]. Therefore, although no significant difference in TAS was observed, its discussion in the manuscript primarily aimed to provide a biochemical context rather than to emphasise a statistically meaningful change. Although similar oxidative stress profiles have been reported in adults [21], the prospective evaluation of these parameters at birth and during the neonatal period represents a unique aspect of our study.

The increase in OSI reflects the predominance of oxidant load relative to antioxidant capacity. Since OSI reveals the biological impact of oxidative stress by evaluating both parameters together, its elevation suggests that ratio-based assessment of redox balance may have clinical significance in the neonatal period. This increase may serve as an early indicator of potential damage in later life.

It has been reported that 21-29% of pregnant women have insufficient B_12_ levels [22]. Since DNA synthesis and cell proliferation accelerate during pregnancy, the requirement for B_12_ increases. The fetal vitamin level is supplied from the mother through the placenta. Deficiency increases the risk of fetal growth restriction, low birth weight, neural tube defects, and preterm birth [23]. Low maternal B_12_ levels have also been associated with increased oxidative stress in the neonatal brain and a higher risk of neurodegenerative diseases [24].

Maternal B_12_ deficiency may also exert long-term effects on fetal epigenetic architecture and developmental programming [25]. Significant associations have been found between maternal B_12_ levels and neonatal DNA methylation profiles. Certain CpG regions have been linked to birth weight and childhood cognitive development [26]. Deficiency may lead to aberrant methylation in genomic imprinting regions [25]. Moreover, it has been reported that increased oxidative DNA damage during the maternal period is paralleled by higher levels in both maternal and cord blood [27].

Taken together, these epigenetic and developmental programming effects support the need for early, objective biomarkers that can capture the long-term impact of intrauterine vitamin B_12_ deficiency.

Studies conducted in our region have shown that maternal B_12_ deficiency is common during pregnancy. In a recent study, deficiency was detected in 73.8% of mothers and 70.5% of newborns, with a strong positive correlation between maternal and neonatal B_12_ levels [28]. Similarly, another study from the same region reported a deficiency in 72% of mothers and 41% of infants, again demonstrating a significant positive correlation between maternal and neonatal levels [29]. These data indicate that B_12_ deficiency in our region is well documented, and therefore re-measuring maternal levels in our study was unnecessary.

Our ROC analysis revealed that 8-OHdG, TOS, and OSI levels had significant diagnostic value in predicting B_12_ deficiency. The observed increase in OSI was primarily driven by elevated TOS levels rather than by decreased TAS, suggesting that the oxidant load played a dominant role in the overall redox imbalance. The highest AUC value was observed for TOS (0.802), followed by 8-OHdG (0.758) and OSI (0.730). These findings may be explained by the fact that B_12_ deficiency increases the oxidant load, influences OSI (the ratio of oxidant burden to antioxidant capacity), and elevates 8-OHdG, a marker of oxidative DNA damage. TAS, on the other hand, had an AUC of 0.409 and was not statistically significant. It is well known that antioxidant defence mechanisms are not yet fully mature in newborns [20]. Although TAS reflects total antioxidant capacity [12], it can be influenced by multiple factors, including maternal-fetal transfer and individual metabolic variability. This may explain the inconsistent results reported in the literature [30]. A systematic review demonstrated that oxidative stress markers, including TOS and OSI, are significantly increased in obstetric complications such as preeclampsia, premature rupture of membranes, and intrauterine growth restriction [31]. Another review noted that methodological and standardisation differences led to inconsistent results, particularly for markers such as TAS [32]. Taken together, these findings suggest that in the neonatal period, TOS, OSI, and 8-OHdG may represent more reliable biomarkers than TAS.

B_12_ deficiency-induced oxidative stress may cause neuronal damage through neuroinflammatory pathways. Microglial activation and increased proinflammatory cytokines accelerate neurodegenerative processes [6]. In this mechanism, the nuclear factor kappa B pathway plays an important role, and its inhibition has been shown to provide neuroprotective effects [33].

Early diagnosis and B_12_ replacement therapy are critical for improving neurological manifestations. B_12_ supplementation can improve cognitive functions; however, its efficacy depends on the duration and severity of the deficiency [34]. It has also been reported that B_12_ supplementation administered during pregnancy and the postpartum period can reduce 8-OHdG levels in both mothers and newborns [10]. Therefore, measuring biomarkers such as 8-OHdG and OSI at birth may help guide diagnostic and therapeutic decisions. From this perspective, our study may contribute to future research by providing biomarker-based guidance for treatment and supporting the determination of reference levels.

Our study is among the few prospective investigations evaluating the impact of vitamin B_12_ deficiency on oxidative stress and DNA damage at birth in healthy term neonates. However, certain limitations should be acknowledged. The relatively small sample size and single-centre design may limit the generali- zability of the findings. Since the study design focused on cord blood, maternal serum levels and urinary 8-OHdG measurements were not performed; therefore, maternal-fetal transfer mechanisms could be inferred only indirectly. Additionally, maternal biochemical and clinical parameters, such as vitamin B_12_, folate, haemoglobin level, maternal age, and supplement use during pregnancy, were not included in the analysis, which represents another limitation of the study. In addition, no additional analyses were conducted regarding other micronutrient deficiencies (e.g., folate, iron). Nevertheless, the study cohort consisted exclusively of healthy term newborns, with evidence of infection or systemic disease excluded, thereby strengthening the reliability of the results.

Vitamin B_12_ deficiency in the neonatal period is associated with a marked increase in oxidative stress and DNA damage. The elevated serum 8-OHdG and OSI levels detected in our study demonstrate that the biological effects of deficiency may begin in the intrauterine period and persist into the postnatal stage. These findings reveal that biomarkers measurable at birth have clinical potential for both diagnosis and long-term risk prediction. Considering the high prevalence of B_12_ deficiency during pregnancy, routine screening, early diagnosis, and timely treatment may play a critical role in preventing haematological, neurodevelopmental, and metabolic adverse outcomes. By providing prospective biomarker data demonstrating oxidative damage associated with B_12_ deficiency, our study provides a solid scientific basis for the development of preventive health strategies targeting the prenatal period.

## Dodatak

### List of abbreviations

8-OHdG, 8-hydroxy-2'-deoxyguanosine;<br>AU, arbitrary units;<br>AUC, area under the curve;<br>CI, confidence interval;<br>C/S, cesarean section;<br>Max, maximum;<br>Min, minimum;<br>NPV negative predictive value;<br>NSVD, normal spontaneous vaginal delivery;<br>OSI, oxidative stress index;<br>PPV, positive predictive value;<br>ROC, receiver operating characteristic;<br>SD, standard deviation;<br>Sens, sensitivity;<br>Spec, specificity;<br>TAS, total antioxidant status;<br>TOS, total oxidant status.

### Acknowledgments

This study did not receive any financial support, donations, or technical assistance.

### Conflict of interest statement

All the authors declare that they have no conflict of interest in this work.
